# The July 2024 Trump assassination attempt was followed by lower in-group support for partisan violence and increased group unity

**DOI:** 10.1073/pnas.2414689121

**Published:** 2024-11-26

**Authors:** Derek E. Holliday, Yphtach Lelkes, Sean J. Westwood

**Affiliations:** ^a^Department of Political Science, Stanford University, Stanford, CA 94305; ^b^Annenberg School of Communication, University of Pennsylvania, Philadelphia, PA 19104; ^c^Department of Government, Dartmouth College, Hanover, NH 03755

**Keywords:** polarization, political violence, partisanship

## Abstract

The attempted assassination of Donald Trump led to widespread concern that the event would escalate political violence between U.S. partisans. While some politicians pleaded for Americans to unite against political violence and “turn down the temperature” on partisan hostility, others continued to engage in inflammatory rhetoric and blame. Using a national survey in the field at the time of the assassination attempt, we take the temperature of America’s partisans before and after the event. We exploit the natural variation induced by the assassination attempt and large daily survey coverage (preattempt: 3,572; postattempt: 703; and 690 in a panel) in the days before and after the attempt to estimate the causal effects of extreme partisan violence on measures of partisan animosity and identity. Using panel and cross-sectional interrupted time series analysis, we find no evidence that the event increased tensions or support for retaliatory violence in the immediate aftermath. On the contrary, Republicans, including MAGA Republicans, became significantly less supportive of partisan violence against Democrats. Republicans also did not become more hostile toward Democrats; instead, their attachment to their own party significantly increased. Democrats experienced no change in attitudes. While nearly a third of Americans have no positive feelings toward the other party, and a supermajority have negative feelings, this animosity was not exacerbated by an extreme but salient instance of partisan violence. Despite the ills of modern political conflict, extreme partisan violence did not cause an immediate upsurge in support for violence.

The attempted assassination of former U.S. president Donald Trump represents a significant escalation in the landscape of political violence in the United States, comparable to the attempted assassination of Ronald Reagan in 1981 and of other heads of state since World War II (1). Assassinations can be massively destabilizing-leading to political breakdowns, economic breakdowns, and worse (1, 2). Understanding the consequence of even failed assassination attempts for partisan attitudes in a highly polarized context like the United States is critical. While the assassination attempt on President Reagan was unifying, with an immediate surge of 11 percentage points in public approval,^*^ the highly polarized modern environment has elevated concerns that the attempt on President Trump has the potential to generate greater partisan hostility and violence (3). We take the temperature of American partisans after the attempted assassination, assessing whether Americans became even more polarized and diagnosing whether this hostility metastasized into support for political violence against opposing partisans.

While many Democratic and Republican politicians encouraged Americans to “turn down the temperature” and appealed to national unity against political violence, others seized the moment to directly blame Democrats for that attack (4). The current literature is somewhat ambivalent on the consequences of extreme partisan violence on the citizenry for both in-group cohesion and a support for out-group hostility. A number of studies suggest that conflict increases in-group cohesion (5), although some work suggests that conflict may reduce in-group cohesion “if the threat has not been properly addressed” (6). Similarly, while some research suggests exposure to political violence begets support for retaliatory actions absent countervailing forces (7), other research suggests experience with violence induces a weariness for hostility and violence (8).

In this paper, we report—using a large daily survey in the field before and after the assassination attempt—causal evidence of the effects of an assassination attempt on public support for partisan violence and on partisan identity. Contrary to a segment of the literature and mass media expectations, our data show that while the attempt increased in-party attachments, support for violence significantly decreased, among Republicans and MAGA Republicans, after the assassination attempt. While Americans hold more negative views of opposing members than ever before (9), an assassination attempt did not inflame tensions.

## Data

We utilize a national survey in the field at the time of the assassination attempt, continuing through the subsequent week. Our analysis focuses on a month: 26 d before the attempt and the 4 d after, with a total sample of 3,572 preattempt and 703 postattempt respondents (out of 104,123 total sample respondents).^†^ In another set of analyses, we examine within-person changes using an embedded panel, where 345 respondents in the postattempt period had been interviewed at least once between September 2022 and June 2024. All respondents were asked a series of questions related to their views of opposing partisans, support for democratic norms, and support for political violence against opposing partisans, identical to those in prior work (10).^‡^

## Increased In-Group Attachment and Decreased Support for Partisan Violence

**Fig. 1.**
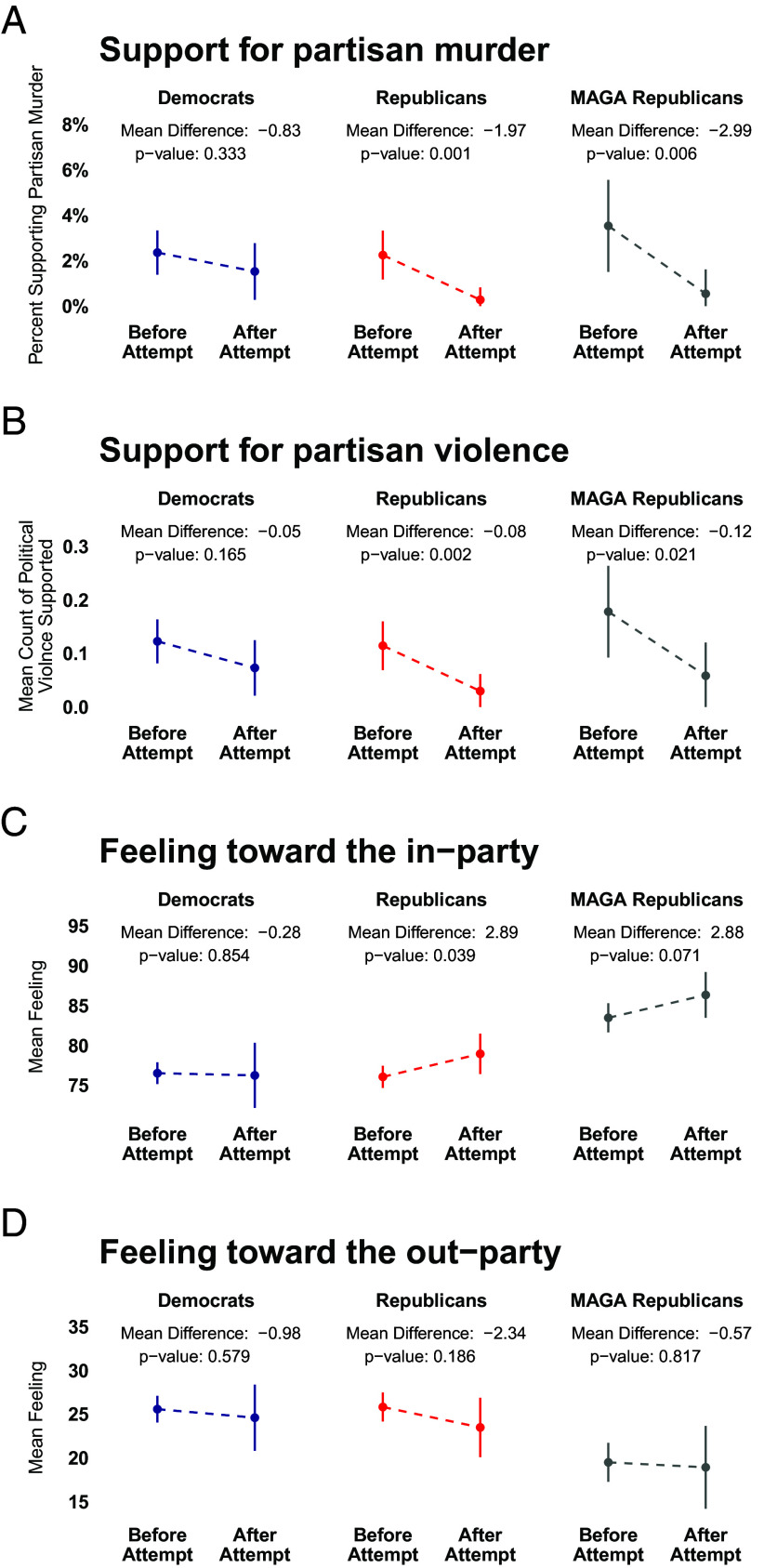
Attitudes before and after the attempted assassination of Donald Trump, by party and MAGA identification, show that Republican support for political violence decreased (*A*, *B*), while in-party affect increased (*C*). There is no significant change in out-party affect (*D*), and no movement among Democrats. Results are weighted to national demographic targets and shown with 95% CIs.

Despite expectations of escalating partisan tensions (11), we found that Republicans became significantly less supportive of partisan violence (Fig. 1*B*) and partisan murder (Fig. 1*A*) in response to the assassination attempt. This decrease was even more pronounced among MAGA Republicans.

The assassination attempt did not make Republicans more hostile toward Democrats. Instead, it increased in-group love among Republicans.^§^

Republican support for the murder of a Democratic party member decreased by just under 2 percentage points to a level indistinguishable from 0, while MAGA support dropped from 3.5 percentage points to a similar level (Fig. 1*A*). Consistent with prior research, baseline support for such extreme violence is low, and it declined across multiple forms of political violence (assault, arson, assault with a deadly weapon, and murder, Fig. 1*B*). We also observed a decrease in the average number of violent acts supported by Republicans (0.08 out of 4), while MAGA support decreased by 0.12.

Rather than demonizing Democrats, Republicans coalesced around their party, with a significant 2.9-point increase in in-party affect (Fig. 1*C*). There was a statistically insignificant decrease in out-party affect (Fig. 1*D*).

Importantly, we also find no significant changes in several related variables. There are no statistically significant increases in the number of democratic norm violations supported (Republicans: −0.12, *P*= 0.1; Democrats: −0.13, *P*= 0.06 on a 0 to 4 scale) or in perceived out-party support for partisan murder (Republicans: −0.4, *P*= 0.86; Democrats: −0.04, *P*= 0.99 on a 0 to 100 scale). This finding is crucial, as prior research suggests that perceived support for violence can serve as a precondition for “preemptive” attacks (12). There was also no significant relationship between affective polarization and postattempt attitude change.

## Robustness

As a robustness test, we constructed every possible window from our full dataset of 104,123 interviews (from 9/16/2022 to 7/12/2024). Of the 637 tests, the largest mean difference in support for partisan murder among Republicans is nearly **50 times smaller** than the difference observed between the pre- and postassassination attempt.

We also found no effects on our outcome measures occur during the comparably salient Democratic National Convention (DNC).

We have balance on covariates in both the pre- and posttreatment samples (age, gender, race, partisanship 7-point, and political interest). All code to reproduce these results is in the replication archive.

## Panel Replication

Our analyses are weighted to national demographic targets, but unobserved heterogeneity between samples may account for some of the variation. To control for time-invariant confounders, we re-estimated the main effects using only respondents with both pre- and postattempt responses, and calculated differences with individual fixed effects.

The panel results are consistent with the cross-sectional results: Support for violence is indistinguishable from zero postattempt, and in-party attachments increase. Importantly, because support for partisan murder is so rare (only 5 panelists preattempt and 4 postattempt), a meaningful difference cannot be estimated with a panel design.

**Fig. 2.**
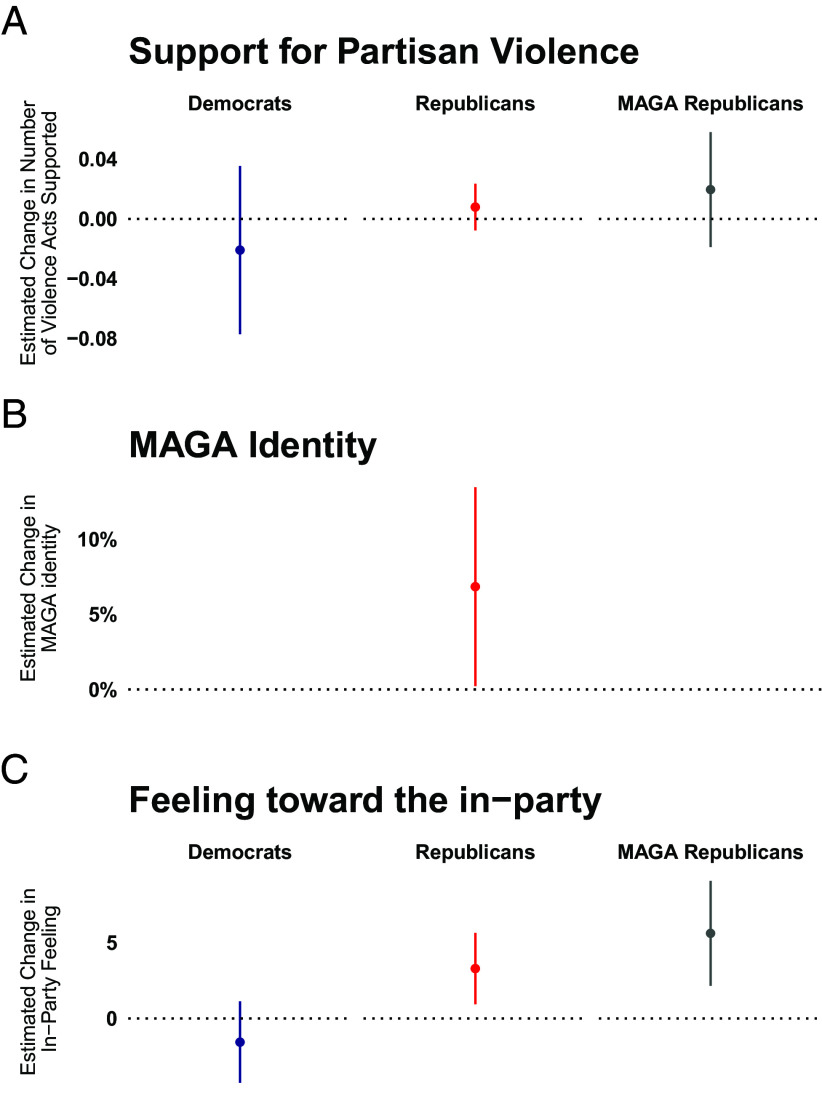
Change in panelist attitudes from before to after the attempted assassination of Donald Trump, by party and pretreatment MAGA identification. Republican MAGA self-identification increased (*B*), as did feelings toward in-party members (*C*). Support for partisan violence remained low and unchanged (*A*). Results are shown with 95% CIs.

We observe significant signs of increased Republican identity for all Republicans and for MAGA Republicans in particular. Postattempt, Republicans were more likely to self-identify as MAGA (Fig. 2*B*) and reported higher in-party feeling (Fig. 2*C*).

These results are not driven by pretreatment trends. As a robustness test, we use July 1, 2024, as a placebo treatment and re-estimate our models. We find no substantive or significant differences on any of our measures.

## Discussion

Previous research offers mixed predictions about the effects of political violence on partisan attitudes. This study demonstrates that exposure to rare (10, 13), extreme acts of violence can promote in-group solidarity without leading to an increase in tolerance for violence against the other party. During this same time, Democrats did not change their attitudes.

Our results have limitations concerning generalizability, construct validity, and internal validity. First, the findings are based on survey responses collected immediately after a specific event. As political activists shape narratives and assign blame, public attitudes may change. Partisans might have responded differently if the assassination had succeeded or if the context had been different (6). Despite using validated measures designed to minimize socially desirable responses (10), the extent to which these survey measures accurately reflect attitudes is always open to debate. The lack of calls for retribution immediately after the attempt suggests that the results have external validity.

Interrupted Time Series designs face several threats to internal validity. The assassination attempt occurred just before the Republican National Convention, although our post-data collection was completed the day before Trump’s speech. While the event and the convention together may represent a bundled treatment, our placebo results suggest that it is unlikely the differences we observed occurred by chance. Additionally, comparing the same set of individuals over time yielded similar results, effectively controlling for selection bias.

In the context of deep partisan tensions, extreme acts of violence do not necessarily deepen partisan divides or perceived threats, but can, under specific conditions, promote unity within political groups and reduce support for violence.

## Materials and Methods

We used survey items from prior work (10). All survey respondents provided informed consent. The study was approved by the respective Institutional Review Boards (IRBs) of Dartmouth College (#STUDY00032563), University of Pennsylvania (851774), and Stanford University (IRB-66755).

## Supplementary Material

Appendix 01 (PDF)

## Data Availability

CSV data have been deposited in OSF (14).

## Footnotes

^*^https://www.washingtonpost.com/archive/politics/1981/04/02/shooting-gives-reagan-boost-in-popularity/9515e340-f295-42e7-89c4-c96ed0ab7a44/.

^†^Our results are robust with 2, 3, and 4 wk pretreatment windows and are not sensitive to the selection of the preattempt bandwidth. Due to the ambiguity of the treatment time, we exclude interviews on 7/13/2024, though results remain unchanged if split at the exact time of the attack.

^‡^We only include partisans in this analysis.

^§^This effect was only marginally significant for MAGA Republicans, who moved from high to even higher levels of in-group love.
