# Supplementary material for: The July 2024 Trump assassination attempt was followed by lower in-group support for partisan violence and increased group unity
Source: Proc Natl Acad Sci U S A. 2024 Nov 26;121(49):e2414689121. doi: 10.1073/pnas.2414689121 (PMC11626138; doi:10.1073/pnas.2414689121)
Supplement: Supplementary file 1 — Appendix 01 (PDF) [file pnas.2414689121.sapp.pdf]

# Supporting Information for

## The July 2024 Trump Assassination Attempt was Followed by Lower In-Group Support for Partisan Violence and Increased Group Unity

**Derek E. Holliday, Yphtach Lelkes, and Sean J. Westwood**

**Sean J. Westwood.**
**E-mail: sean.j.westwood@dartmouth.edu**

**This PDF file includes:**

Supporting text

## Supporting Information Text

## Full Question Wording

Below we include the full question text of the items underlying out estimates, by section. All sections are presented in random order, with the exception of political violence perceptions, which always follow the political violence section. All demographic items were gathered separately by YouGov.

**A. Affect and Trust.** Order within this section is completely randomized.

- **democrat_therm:** We'd like you to rate how you feel towards some groups on a scale of 0 to 100. Zero means very unfavorable and 100 means very favorable. Fifty means you do not feel favorable or unfavorable. How would you rate your feeling toward Democrats? [Response: 0-100 thermometer]
- **republican_therm:** We'd like you to rate how you feel towards some groups on a scale of 0 to 100. Zero means very unfavorable and 100 means very favorable. Fifty means you do not feel favorable or unfavorable. How would you rate your feeling toward Republicans? [Response: 0-100 thermometer]
- **maga:** Would you describe yourself as a... [Response: MAGA Republican / Never Trumper / Neither]

**B. Democratic Norms.** Order within this section is randomized. Response options for the norms questions are always Strongly agree / Agree / Neither agree nor disagree / Disagree / Strongly disagree.

- **norm_polling:** Do you agree or disagree: (inparty) should reduce the number of polling stations in areas that typically support (outparty).
- **norm_loyalty:** Do you agree or disagree with the following: When a (inparty) candidate questions the outcome of an election other (inparty) should be more loyal to the (inparty) party than to election rules and the constitution.
- **norm_judges:** Do you agree or disagree: (inparty) elected officials should sometimes consider ignoring court decisions when the judges who issued those decisions were appointed by (outparty) presidents.
- **norm_censorship:** Do you agree or disagree with the following: The government should be able to censor media sources that spend more time attacking (inparty) than (outparty).

**C. Political Violence.** The questions in this section appear in the order presented below. For all items, the response options are Strongly support / Support / Neither support nor oppose / Oppose / Strongly oppose.

- **violence1:** (name) was convicted of protesting without a permit. He was arrested by police after leading a protest against (outparty) on the grounds of the county courthouse. He made no effort to acquire the necessary permit for the protest and refused to leave when asked by police. Do you support or oppose (name)'s actions?
- **violence2:** (name) was convicted of vandalism. He was arrested by police after he vandalized several large signs expressing support for candidates of the (outparty) party. Do you support or oppose (name)'s actions?
- **violence3:** (name) was convicted of assault. He was arrested by police for throwing rocks at peaceful (outparty) protesters. Although no one was seriously injured, paramedics bandaged a man with a head wound. Do you support or oppose (name)'s actions?
- **violence4:** (name) was convicted of arson. He was arrested by police as he attempted to run from a fire he started at the local (outparty) headquarters. Although he waited for the building to close for the night, several adjacent buildings were still occupied. Do you support or oppose (name)'s actions?
- **violence5:** (name) was convicted of assault with a deadly weapon. He was arrested by police after driving his car into a crowd of (outparty) protesters. Although no one was killed, several individuals were seriously injured and one spent a month in the hospital. Do you support or oppose (name)'s actions?
- **violence6** (name) was convicted of murder. He was arrested by police after surveillance footage was found showing him stabbing a prominent (outparty) to death. (name) targeted the victim because he believed the victim had prevented him from voting in the last election as part of a conspiracy to stop (inparty) voters. Do you support or oppose (name)'s actions?

**D. Political Violence Perceptions.** The response option for the following item was a 0-100 slider.

- **violence6_perception:** (name) was convicted of murder. He was arrested by police after surveillance footage was found showing him stabbing a prominent (inparty) to death. (name) targeted the victim because he believed the victim had prevented him from voting in the last election as part of a conspiracy to stop (outparty) voters. What percent of (outparty) voters do you think support (name)'s actions?

## Sample Properties

The data from this study comes YouGov. The population under study was the adult U.S. population. YouGov employs a two-step process to conduct surveys. Initially, they select a random set of anonymous cases from the American Community Survey (ACS) Public Use Microdata Sample, a reliable data source which accurately represents various variables in the target population. This subset is known as the synthetic sampling frame (SSF) and serves as a model for the final survey sample. For each participant who takes the YouGov survey, they find a matching case within the SSF that shares similar characteristics.

Specifically, YouGov constructs its sampling frame based upon ACS targest for age, gender, race, and education, with weights constructed using propensity scores.* YouGov uses nonprobability quota sampling to achieve representativeness, and due to the opt-in design and wide availability of YouGov surveys across many online channels, it is impossible to provide survey response rates.

YouGov respondents are weighted to nationally representative targets for age, gender, race, education, region, political party affiliation, and income level using propensity scores. Large weights are trimmed. Final weights are normalized to sample size.

## Extended Methods

Our main analysis is a simple ordinary least squares regression:

$$y_i = \alpha + \text{post-attempt}_i \beta + \epsilon_i,$$

where $y_i$ is the dependent variable (support for partisan violence, MAGA identity, and in-party feeling thermometers) for individual $i$, $\alpha$ is a global intercept, and post-attempt is a binary indicator for whether the response takes place after the assassination attempt. We estimate this model separately for Democrats, Republicans, and self-identified MAGA Republicans.

For the panel analysis, we estimated a simple one-way fixed effect panel model with individual-specific intercepts, with the following form:

$$y_{it} = \alpha_i + \text{post-attempt}_{it} \beta + \epsilon_{it},$$

where $\alpha_i$ is the individual-level fixed effect for the panelist, and we observe our dependent variable at time $t$. We subset our panelist data to only the most recent pre-attempt response and post-attempt response.

* See https://today.yougov.com/about/panel-methodology
